# Supplementary material for: WWOX Loses the Ability to Regulate Oncogenic AP-2γ and Synergizes with Tumor Suppressor AP-2α in High-Grade Bladder Cancer
Source: Cancers (Basel). 2021 Jun 12;13(12):2957. doi: 10.3390/cancers13122957 (PMC8231628; doi:10.3390/cancers13122957)
Supplement: Supplementary file 1 [file cancers-13-02957-s001.zip › Table S1.pdf]

Table S1. Functional annotation of DEGs in specific comparisons

Comparison	Profile	Biological process (from PANTHER GO-Slim)	Gene(s)	*p-value*
**CAL-29 KA vs. KK**	downregulated	Apoptotic mitochondrial changes	*HSPD1*<br>*BNIP3L*<br>*DNM1L*	7.47E-04
		Cell adhesion mediated by integrin	*ITGB6*<br>*ITGB1*	1.85E-02
		Cell-cell adhesion	*HSPD1*<br>*DSG2*<br>*ANXA1*<br>*DSG3*<br>*TJP1*<br>*LPP*	3.21E-02
		Apoptotic cleavage of cell adhesion proteins	*DSG2*<br>*DSG3*<br>*TJP1*	7.47E-04
		Downregulation of ERBB4 signaling	*ITCH*<br>*NEDD4*	7.31E-03
		Regulation of activated PAK-2p34 by proteasome mediated degradation	*PSMD12*<br>*PSMD14*<br>*PSMC2*<br>*PSMD1*	5.27E-03
		Negative regulation of NOTCH4 signaling	*PSMD12*<br>*PSMD14*<br>*PSMC2*<br>*PSMD1*	6.41E-03
		Regulation of RUNX2 expression and activity	*PSMD12*<br>*PSMD14*<br>*STAT1*<br>*PSMC2*<br>*PSMD1*	3.17E-03
		Regulation of RAS by GAPs	*PSMD12*<br>*PSMD14*<br>*PSMC2*<br>*PSMD1*	1.39E-02
	upregulated	Cell migration	*CCL2*<br>*TNFAIP3*<br>*CXCL8*<br>*S100A9*<br>*CXCL1*	1.08E-03
		Positive regulation of Wnt signaling pathway	*TNFAIP3*<br>*SULF2*	3.43E-03
		Mitotic cell cycle	*CKS2*<br>*CENPW*<br>*PTTG1*	4.38E-02
**CAL-29 KC vs. KK**	downregulated	Blood vessel morphogenesis	*EFNB2*<br>*THBS1*	4.78E-02
		Negative regulation of cell death	*EYA2*<br>*AGR2*<br>*APOE*	2.74E-02
		Downregulation of TGF-beta receptor signaling	*TGFBR2*<br>*PMEPA1*<br>*BAMBI*	1.02E-03
		Integrin cell surface interactions	*COL5A2*<br>*COL5A1*	3.12E-07

			COL4A2<br>*COL4A1*<br>*FN1*<br>*COL7A1*<br>*THBS1*<br>*TNC*	
		Collagen degradation	*COL5A2*<br>*COL5A1*<br>*COL4A2*<br>*COL4A1*<br>*COL7A1*	1.34E-04
	upregulated	Regulation of cell cycle	*NUPR1*<br>*HBEGF*<br>*PTTG1*<br>*PLK1*<br>*EREG*	2.16E-02
		ERBB signaling pathway	*HBEGF*<br>*EREG*	1.49E-02
		Regulation of cell growth	*SLIT2*<br>*SEMA5A*	2.66E-02
		Extracellular matrix organization	*MME*<br>*LAMB4*<br>*ADAMTSL4*<br>*SMOC1*<br>*MMP28*	2.16E-03
		Cell migration	*CXCL3*<br>*LAMB4*<br>*RHOF*<br>*S100A9*<br>*SEMA5A*<br>*S100A14*	1.58E-02
		G2/M DNA damage checkpoint	*H2BC13*<br>*H2BC5*<br>*H2BC11*<br>*H2BC17*<br>*H2BC15*<br>*H2BC21*	3.96E-05
		Oncogene Induced Senescence	*CDKN2A*<br>*CDKN2B*	2.81E-02
		Degradation of the extracellular matrix	*MME*<br>*FBN2*<br>*FBN1*<br>*CAPN8*	2.30E-02
**CAL-29 WK vs. KK**	downregulated	Programmed cell death	*HSPD1*<br>*CASP4*<br>*ANXA1*<br>*DDIT4*<br>*BNIP3*<br>*BEX2*<br>*BNIP3L*<br>*HERPUD1*<br>*DDIT3*<br>*HYOU1*	1.06E-03
		Positive regulation of angiogenesis	*ANXA1*<br>*VEGFA*	9.34E-03

		Negative regulation of intrinsic apoptotic signaling pathway	*HERPUD1*<br>*HYOU1*	9.34E-03
		Positive regulation of mitotic cell cycle	*ANXA1*<br>*AREG*	3.27E-02
		Apoptotic cleavage of cell adhesion proteins	*DSG3*<br>*OCLN*	1.10E-02
		Negative regulation of NOTCH4 signaling	*PSMD14*<br>*PSMC1*<br>*PSMD1*	3.09E-02
		RAF/MAP kinase cascade	*ARTN*<br>*PSMD14*<br>*PSMC1*<br>*MET*<br>*DUSP4*<br>*FN1*<br>*PSMD1*<br>*AREG*	1.05E-02
	upregulated	Response to tumor necrosis factor	*CCL2*<br>*UBD*<br>*CX3CL1*	1.53E-02
		Positive regulation of cell migration	*SEMA3F*<br>*S100A14*<br>*CLEC7A*	3.12E-02
		Negative regulation of mitotic cell cycle	*BTG2*<br>*ZWINT*<br>*PTTG1*	3.40E-02
		Positive regulation of apoptotic process	*PYDC1*<br>*UBD*<br>*DAPK1*	3.69E-02
		Cell population proliferation	*TBX3*<br>*BTG2*<br>*LYN*<br>*STAT6*<br>*CDK2*<br>*PLA2G2A*<br>*S100A13*<br>*CLEC7A*	2.26E-03
		G2/M DNA damage checkpoint	*H2BC12*<br>*H2BC4*<br>*H2BC13*<br>*H2BC5*<br>*H2BC11*<br>*H2BC9*<br>*H2BC14*<br>*H2BC3*<br>*H2AX*<br>*H2BC17*<br>*H4C9*<br>*H4C13*<br>*H4C1*<br>*H4C4*<br>*H2BC15*<br>*H2BC21*	4.36E-11
**CAL-29 WA vs. WK**	downregulated	Regulation of Notch signaling pathway	*GALNT11*<br>*DLL1*	7.92E-03
		Cell Cycle, Mitotic	*H2BC12*	2.76E-14

	upregulated		*H2AC7*<br>*POLA2*<br>*H2BC4*<br>*LYN*<br>*H2BC13*<br>*H2AC20*<br>*LIG1*<br>*H2BC5*<br>*H2BC11*<br>*TYMS*<br>*H2AC14*<br>*INCENP*<br>*H2BC9*<br>*H2BC14*<br>*H2BC3*<br>*FOXM1*<br>*H2BC17*<br>*H3C12*<br>*H4C9*<br>*H4C13*<br>*H4C1*<br>*H4C4*<br>*H4C3*<br>*H2BC15*<br>*H2BC21*<br>*H2AC8*<br>*CENPM*	
		G2/M DNA damage checkpoint	*H2BC12*<br>*H2BC4*<br>*H2BC13*<br>*RMI2*<br>*H2BC5*<br>*H2BC11*<br>*H2BC9*<br>*H2BC14*<br>*H2BC3*<br>*H2BC17*<br>*H4C9*<br>*H4C13*<br>*H4C1*<br>*H4C4*<br>*H4C3*<br>*H2BC15*<br>*H2BC21*	2.15E-14
	upregulated	Positive regulation of mitotic cell cycle	*HBEGF*<br>*ANXA1*<br>*AREG*<br>*EREG*	5.13E-05
		Regulation of apoptotic signaling pathway	*HERPUD1*<br>*HYOU1*	3.43E-02
		Regulation of epidermal growth factor receptor signaling pathway	*HBEGF*<br>*AREG*<br>*EREG*	5.83E-04
		Wound healing	*ANXA1*<br>*ANXA6*	3.61E-02
		Positive regulation of cell population proliferation	*HBEGF*<br>*ANXA1*	7.93E-03

			AREG<br>*EREG*	
		Extracellular matrix organization	*COL5A1*<br>*MMP13*<br>*COL4A2*<br>*SULF2*	1.81E-02
**CAL-29 WC vs. WK**	downregulated	Negative regulation of mitotic cell cycle	*BTG2*<br>*RBL2*	3.29E-02
		Apoptotic process	*CD74*<br>*PYDC1*<br>*UBD*<br>*CRIP1*	2.56E-02
		Negative regulation of cell population proliferation	*BTG2*<br>*LGALS9*<br>*PLA2G2A*	8.32E-04
		Negative regulation of MAP kinase activity	*DUSP2*<br>*DUSP1*	3.99E-03
		Positive regulation of apoptotic process	*PYDC1*<br>*UBD*	3.49E-02
	upregulated	Positive regulation of mitotic cell cycle	*HBEGF*<br>*RANBP1*<br>*AREG*	1.75E-03
		Cell population proliferation	*CAV2*<br>*HBEGF*<br>*ZPR1*<br>*TGFB2*<br>*RTKN2*<br>*MYC*<br>*CAV1*<br>*S100A13*<br>*AREG*	1.97E-04
		Transforming growth factor beta receptor signaling pathway	*TGFB2*<br>*PMEPA1*<br>*ZYX*	4.82E-03
		ERBB signaling pathway	*HBEGF*<br>*AREG*	2.61E-02
		Mitotic cell cycle process	*CCNB1*<br>*TUBB*<br>*HBEGF*<br>*CKS2*<br>*CENPW*<br>*TUBA4A*<br>*TUBB2A*<br>*TUBB4B*<br>*RANBP1*<br>*RTKN2*<br>*MAD2L1*<br>*TUBA1A*<br>*TUBA1B*<br>*SKA1*<br>*PLK1*<br>*TUBB3*<br>*TUBA1C*<br>*AREG*	7.44E-10
		Extracellular matrix organization	*HAS3*<br>*COL5A1*	2.55E-04

			MMP13<br>*MMP9*<br>*COL4A2*<br>*COL4A1*<br>*SULF2*	
		Positive regulation of cell population proliferation	*HBEGF*<br>*RTKN2*<br>*MYC*<br>*S100A13*<br>*AREG*	2.78E-03
		Wnt signaling pathway	*DKK1*<br>*DKK3*<br>*WNT4*<br>*SULF2*	4.00E-02
		Degradation of the extracellular matrix	*COL5A1*<br>*LAMC2*<br>*KLK7*<br>*MMP13*<br>*MMP9*<br>*COL4A2*<br>*COL4A1*	7.47E-04
**HT-1376 KA vs. KK**	downregulated	Endothelial cell migration	*S100P*	6.78E-03
		Cell death signalling via NRAGE, NRIF and NADE	*MCF2L*	3.68E-02
		Crosslinking of collagen fibrils	*LOXL4*	9.18E-03
		Pre-NOTCH Expression and Processing	*ELF3*	3.77E-02
		Extracellular matrix organization	*ELF3*<br>*LOXL4*	1.28E-02
		Positive regulation of apoptotic process	*TXNIP*<br>*MCF2L*	2.89E-02
		Transmembrane receptor protein tyrosine kinase signaling pathway	*TXNIP*<br>*ATP6V1B1*	2.56E-02
	upregulated	Cell migration	*CCL2*<br>*FSCN1*<br>*TUBB2A*	1.28E-02
		p130Cas linkage to MAPK signaling for integrins	*FN1*	2.91E-02
		Integrin binding	*CCN1*<br>*FN1*	2.11E-03
		Regulation of cell population proliferation	*CCL2*<br>*EGR1*<br>*TGM2*<br>*MAL*<br>*FGFBP1*<br>*IL24*<br>*SGK1*<br>*CCN1*<br>*SERPINE2*<br>*DHRS2*<br>*DDAH1*<br>*FN1*<br>*FOSL1*	5.12E-06
		Angiogenesis	*CCL2*<br>*CCN1*<br>*FN1*	2.19E-02
		Regulation of cell adhesion	*CCL2*<br>*TGM2*<br>*CCN1*	1.02E-02

			SERPINE2<br>*FN1*	
		Regulation of growth	*SGK1*<br>*STC2*<br>*SERPINE2*<br>*MT1G*<br>*FN1*	7.82E-03
		Positive regulation of apoptotic process	*CCL2*<br>*TGM2*<br>*FOSL1*<br>*MAL*	1.96E-02
		Positive regulation of I-kappaB kinase/NF-kappaB signaling	*TGM2*<br>*MAL*	4.69E-02
		Extracellular matrix organization	*CCN1*<br>*KLK5*<br>*FN1*	2.95E-02
		Regulation of cell growth	*SGK1*<br>*SERPINE2*<br>*FN1*	4.23E-02
**HT-1376 KC vs. KK**	downregulated	Glial cell proliferation	*CLU*	2.42E-02
		Positive regulation of fibroblast apoptotic process	*BTG1*	1.22E-02
		Regulation of cell population proliferation	*CLU*<br>*BTG1*<br>*HTRA1*<br>*ATOH8*<br>*ZNF703*<br>*CTSH*	2.25E-02
		Regulation of transforming growth factor beta receptor signaling pathway	*HTRA1*<br>*ZNF703*	1.24E-02
		Positive regulation of cell migration	*ATOH8*<br>*ZNF703*<br>*CTSH*	3.37E-02
	upregulated	Regulation of cell-cell adhesion	*CD74*<br>*ADAM8*<br>*CCL2*<br>*TNF*<br>*CD70*<br>*ANXA1*<br>*PODXL*<br>*LGALS1*<br>*TGFB1*<br>*PYCARD*<br>*ICAM1*<br>*IGFBP2*<br>*CEBPB*<br>*ZC3H12A*	1.70E-07
		Regulation of cell migration	*CD74*<br>*ADAM8*<br>*CCL2*<br>*GLIPR2*<br>*TNF*<br>*ANXA1*<br>*LAMA3*<br>*PODXL*<br>*GAS6*<br>*IL24*	4.76E-09

			ITGB1<br>*MYLK*<br>*TGFB1*<br>*S100A7*<br>*PYCARD*<br>*ICAM1*<br>*HIF1A*<br>*SOD2*<br>*CEMIP*<br>*ZC3H12A*<br>*TGFBR1*<br>*SEMA3C*	
		Negative regulation of endothelial cell-matrix adhesion via fibronectin	*MME*	1.10E-02
		Negative regulation of cell adhesion	*CD74*<br>*MME*<br>*ANXA1*<br>*PODXL*<br>*LGALS1*<br>*TGFB1*<br>*CEBPB*<br>*ZC3H12A*	2.15E-04
		Negative regulation of apoptotic process	*CD74*<br>*ADAM8*<br>*CCL2*<br>*TNF*<br>*ANXA1*<br>*GAS6*<br>*ITGB1*<br>*CHST11*<br>*IER3*<br>*ICAM1*<br>*SERPINB2*<br>*HIF1A*<br>*SOD2*<br>*DHRS2*<br>*SOCS3*<br>*FCMR*<br>*CEBPB*<br>*MT1G*<br>*ZC3H12A*<br>*TGFBR1*<br>*ITGA5*<br>*PLK3*	6.78E-09
		Cell migration	*CD74*<br>*ADAM8*<br>*MME*<br>*S100A2*<br>*CCL2*<br>*CXCL3*<br>*TNF*<br>*FSCN1*<br>*ANXA1*<br>*SAA1*<br>*LAMA3*<br>*PODXL*<br>*GAS6*	1.44E-10

			TUBB2A<br>*ITGB1*<br>*TGFB1*<br>*ANXA6*<br>*S100A9*<br>*ICAM1*<br>*HIF1A*<br>*S100A8*<br>*PSG1*<br>*TGFBR1*<br>*SEMA3C*<br>*ITGA5*	
		Wound healing	*MME*<br>*SERPINA1*<br>*SAA1*<br>*GAS6*<br>*IL24*<br>*TGFB1*<br>*ANXA6*<br>*SERPINB2*<br>*HIF1A*<br>*S100A8*<br>*TGFBR1*<br>*ITGA5*	7.30E-06
		Cell adhesion mediated by integrin	*ITGB1*<br>*ICAM1*<br>*ITGA5*	4.32E-04
		angiogenesis	*ADAM8*<br>*CCL2*<br>*TYMP*<br>*SAT1*<br>*ITGB1*<br>*S100A7*<br>*COL8A1*<br>*HIF1A*<br>*ZC3H12A*<br>*TGFBR1*<br>*ITGA5*	2.39E-06
		Regulation of extracellular matrix organization	*FSCN1*<br>*TGFB1*	2.44E-02
		Positive regulation of programmed cell death	*ADAM8*<br>*CCL2*<br>*TNF*<br>*UBD*<br>*TGM2*<br>*ANXA1*<br>*ITGB1*<br>*TGFB1*<br>*S100A9*<br>*INHBA*<br>*PYCARD*<br>*SOD2*<br>*S100A8*<br>*ZC3H12A*<br>*TGFBR1*	7.38E-07
		Regulation of epithelial to mesenchymal transition	*GLIPR2*<br>*TGFB1*	1.56E-02

			TGFBR1	
		Regulation of intrinsic apoptotic signaling pathway	*CD74*<br>*S100A9*<br>*PYCARD*<br>*HIF1A*<br>*SOD2*<br>*S100A8*	3.48E-04
		Positive regulation of SMAD protein signal transduction	*TGFB1*<br>*TGFBR1*	4.34E-03
		Extrinsic apoptotic signaling pathway	*TNF*<br>*CD70*<br>*TGFB1*<br>*INHBA*<br>*LCN2*<br>*IFI27*	2.39E-05
		Negative regulation of anoikis	*ITGB1*<br>*ITGA5*	5.35E-03
		Positive regulation of NF-kappaB transcription factor activity	*ADAM8*<br>*TNF*<br>*TGFB1*<br>*S100A9*<br>*PYCARD*<br>*ICAM1*<br>*S100A8*	4.37E-05
		Negative regulation of tumor necrosis factor production	*GAS6*<br>*ZC3H12A*	4.07E-02
		Activation of MAPK activity	*CD74*<br>*TNF*<br>*SAA1*<br>*INAVA*	1.19E-02
		Regulation of cell growth	*TGFB1*<br>*S100A9*<br>*INHBA*<br>*S100A8*<br>*TGFBR1*<br>*SEMA3C*	3.02E-02
**HT-1376 WK vs. KK**	downregulated	Positive regulation of epithelial to mesenchymal transition	*TGFB2*<br>*ZNF703*<br>*CTNNB1*	6.55E-03
		Regulation of programmed cell death	*TNFSF10*<br>*CLU*<br>*MTRNR2L2*<br>*MTRNR2L12*<br>*TXNIP*<br>*PPIA*<br>*FHL2*<br>*MTRNR2L8*<br>*DAPK1*<br>*UBC*<br>*PPT1*<br>*PLAC8*<br>*SCIN*<br>*TGFB2*<br>*SON*<br>*CRYAB*<br>*HPGD*	4.02E-08

			ADAMTSL4<br>*BEX2*<br>*LDHA*<br>*TIMP3*<br>*CTNNB1*<br>*EIF2AK4*<br>*CITED2*<br>*PDIA3*<br>*NONO*<br>*SEMA5A*<br>*PARP1*<br>*SKP2*<br>*PLAC8*	
		Negative regulation of cell cycle	*ZNF207*<br>*RBBP4*<br>*TGFB2*<br>*HPGD*<br>*SKP1*<br>*CHMP4C*<br>*CTNNB1*<br>*EIF2AK4*<br>*TFDP2*<br>*HSP90AB1*	1.28E-02
		Transforming growth factor beta receptor signaling pathway	*UBC*<br>*TGFBR3*<br>*TGFB2*<br>*HPGD*<br>*CITED2*<br>*PARP1*	1.38E-04
		Regulation of cell cycle process	*PRC1*<br>*TPX2*<br>*KIF20A*<br>*ZNF207*<br>*RACGAP1*<br>*RBBP4*<br>*ADAMTS1*<br>*HNRNPU*<br>*CALM1*<br>*SKP1*<br>*CHMP4C*<br>*CTNNB1*<br>*TFDP2*<br>*HSP90AB1*	2.23E-03
		Positive regulation of cell population proliferation	*PRC1*<br>*ADAMTS1*<br>*HTRA1*<br>*HNRNPU*<br>*TGFBR3*<br>*TGFB2*<br>*HPGD*<br>*ZNF703*<br>*CTNNB1*<br>*SEMA5A*<br>*CD55*<br>*SKP2*<br>*PLAC8*	3.24E-02
		Negative regulation of cell cycle process	*ZNF207*	4.02E-02

			RBBP4<br>*SKP1*<br>*CHMP4C*<br>*TFDP2*<br>*HSP90AB1*	
		Cell growth	*SEMA3F*<br>*CTNNB1*<br>*EIF2AK4*<br>*SEMA5A*<br>*HSP90AB1*	8.09E-04
		Regulation of intrinsic apoptotic signaling pathway	*CLU*<br>*PPIA*<br>*NONO*<br>*PARP1*	3.68E-02
		Positive regulation of cell size	*PRR16*<br>*HSP90AB1*	4.76E-03
		Positive regulation of transforming growth factor beta receptor signaling pathway	*TGFBR3*<br>*CITED2*<br>*HSP90AB1*	1.73E-03
		Extracellular structure organization	*COL12A1*<br>*ADAMTS1*<br>*HTRA1*<br>*LAMB4*<br>*TGFB2*<br>*ADAMTSL4*<br>*FBN2*<br>*FBN1*<br>*LOXL4*	2.00E-03
		Wnt signaling pathway, calcium modulating pathway	*CALM1*<br>*CTNNB1*	3.27E-02
		Negative regulation of cell population proliferation	*RBBP4*<br>*ADAMTS1*<br>*SCIN*<br>*ATP5F1A*<br>*TGFBR3*<br>*TGFB2*<br>*ATOH8*<br>*P3H2*<br>*CTNNB1*<br>*IGFBP7*<br>*CITED2*<br>*ASCL2*	1.16E-02
		Positive regulation of epithelial cell migration	*ATP5F1A*<br>*TGFB2*<br>*ATOH8*<br>*SEMA5A*	2.97E-02
		Angiogenesis	*GJA5*<br>*TGFBR3*<br>*THSD7A*<br>*CTNNB1*<br>*CLIC4*<br>*SEMA5A*	3.74E-02
	upregulated	Negative regulation of MAPK cascade	*DUSP2*<br>*DUSP6*<br>*LIF*<br>*TNIP1*	2.28E-03

			DUSP4<br>*DUSP7*<br>*EPHB2*	
		Negative regulation of apoptotic signaling pathway	*BID*<br>*CD74*<br>*PLAUR*<br>*TNFAIP3*<br>*CSF2*<br>*WNT4*<br>*MMP9*<br>*THBS1*	2.93E-03
		Negative regulation of meiotic cell cycle	*OVOL1*<br>*LIF*	1.51E-02
		Negative regulation of cell migration	*CD74*<br>*CARD10*<br>*ADGRG1*<br>*IL24*<br>*IGFBP3*<br>*WNT4*<br>*KRT16*<br>*ALOX15B*<br>*THBS1*	2.03E-03
		Regulation of extracellular matrix organization	*SOX9*<br>*FSCN1*<br>*TNFRSF1B*	1.01E-02
		Regulation of angiogenesis	*TNFAIP3*<br>*VEGFA*<br>*HMGA2*<br>*C3*<br>*CXCL8*<br>*TWIST1*<br>*ALOX5*<br>*ZC3H12A*<br>*THBS1*<br>*F3*<br>*NMB*	1.84E-04
		Negative regulation of sprouting angiogenesis	*ALOX5*<br>*THBS1*	7.15E-03
		Negative regulation of MAP kinase activity	*DUSP2*<br>*DUSP6*<br>*DUSP4*<br>*DUSP7*	8.73E-03
		Positive regulation of extrinsic apoptotic signaling pathway	*BID*<br>*INHBA*<br>*PYCARD*<br>*G0S2*<br>*THBS1*<br>*MAL*	2.15E-05
		Negative regulation of cell migration involved in sprouting angiogenesis	*CARD10*<br>*THBS1*	1.21E-02
		Mesenchymal to epithelial transition	*ALOX5*<br>*ALOX15B*	1.36E-02
		Intrinsic apoptotic signaling pathway	*DDIT4*<br>*CDKN1A*<br>*TNFRSF1B*<br>*PYCARD*	4.92E-03

			IKBKE<br>*CEBPB*	
		Positive regulation of extracellular matrix organization	*LIF*<br>*INHBA*	2.36E-02
		Extracellular matrix disassembly	*ADAM8*<br>*MMP2*<br>*KLK7*<br>*MMP9*<br>*KLK5*	7.11E-04
		Extrinsic apoptotic signaling pathway	*BID*<br>*CD70*<br>*TNFRSF1B*<br>*INHBA*<br>*LCN2*<br>*G0S2*<br>*IFI27*	8.71E-05
		Extracellular matrix assembly	*HAS3*<br>*COL1A2*	4.49E-02
		Positive regulation of angiogenesis	*VEGFA*<br>*HMGA2*<br>*C3*<br>*CXCL8*<br>*TWIST1*<br>*ZC3H12A*<br>*THBS1*<br>*F3*	2.64E-04
		Positive regulation of tumor necrosis factor production	*MAL*<br>*PYCARD*<br>*TWIST1*<br>*THBS1*	1.20E-02
		Positive regulation of NF-kappaB transcription factor activity	*ADAM8*<br>*MAL*<br>*IRAK2*<br>*NFKB2*<br>*S100A9*<br>*PYCARD*<br>*S100A8*	1.60E-03
		Cell cycle arrest	*CD74*<br>*MAL*<br>*VEGFA*<br>*S100A7*<br>*JUN*<br>*PYCARD*<br>*NMB*	5.86E-03
		Negative regulation of epithelial cell proliferation	*SOX9*<br>*EFNB2*<br>*ALOX5*<br>*THBS1*	4.71E-02
**HT-1376 WA vs. WK**	downregulated	Negative regulation of growth hormone receptor signaling pathway	*GDF15*	8.74E-04
		Extrinsic apoptotic signaling pathway	*LCN2*	2.85E-02
		SMAD protein signal transduction	*GDF15*	1.82E-02
		Transforming growth factor beta receptor signaling pathway	*GDF15*	2.88E-02
		Positive regulation of MAPK cascade	*S100A7*<br>*GDF15*	9.18E-03

	upregulated	Negative regulation of apoptotic process	*CCL2*<br>*UBC*<br>*GAS6*<br>*CTNNB1*<br>*MT1G*<br>*THBS1*	2.57E-03
		Regulation of cell population proliferation	*CCNB1*<br>*CCL2*<br>*GAS6*<br>*ATP5F1A*<br>*TPM1*<br>*CTNNB1*<br>*GSDME*<br>*THBS1*<br>*TNC*	7.56E-04
		Canonical Wnt signaling pathway involved in positive regulation of epithelial to mesenchymal transition	*CTNNB1*	4.65E-03
		Negative regulation of cell cycle	*CCNB1*<br>*CCL2*<br>*CTNNB1*<br>*THBS1*	1.19E-02
		Canonical Wnt signaling pathway involved in negative regulation of apoptotic process	*CTNNB1*	6.19E-03
		Negative regulation of cell population proliferation	*CCL2*<br>*ATP5F1A*<br>*TPM1*<br>*CTNNB1*<br>*GSDME*<br>*THBS1*	6.56E-04
		Regulation of apoptotic signaling pathway	*CTNNB1*<br>*PARP1*<br>*GSDME*<br>*THBS1*	2.30E-03
		Positive regulation of apoptotic process	*CCL2*<br>*UBC*<br>*CTNNB1*<br>*GSDME*<br>*THBS1*	1.65E-03
		Negative regulation of angiogenesis	*CTNNB1*<br>*THBS1*	1.19E-02
		Mitotic cell cycle process	*CCNB1*<br>*CCNJL*<br>*RAN*<br>*STMN1*	2.01E-02
		Positive regulation of immune response to tumor cell	*GSDME*	2.30E-02
		Positive regulation of SMAD protein signal transduction	*PARP1*	2.61E-02
		Angiogenesis	*CCL2*<br>*PRCP*<br>*CTNNB1*<br>*THBS1*	1.57E-03
		Necrotic cell death	*GSDME*	4.70E-02
		Extracellular matrix organization	*GAS6*<br>*THBS1*<br>*TNC*	1.88E-02

		Negative regulation of G1/S transition of mitotic cell cycle	*CCNB1*<br>*CCL2*	1.24E-02
		Positive regulation of MAPK cascade	*CCL2*<br>*UBC*<br>*GAS6*<br>*CTNNB1*<br>*GSDME*<br>*CAP1*<br>*THBS1*	1.42E-05
**HT-1376 WC vs. WK**	downregulated	Positive regulation of apoptotic signaling pathway	*CTSC*	1.85E-02
		Cell killing	*CTSC*	1.74E-02
		Positive regulation of inflammatory response	*CTSC*	2.04E-02
		Negative regulation of myelination	*CTSC*	1.16E-03
	upregulated	Negative regulation of apoptotic process	*CLU*<br>*MTRNR2L2*<br>*MTRNR2L12*<br>*MTRNR2L8*<br>*GAS6*	7.20E-03
		Negative regulation of execution phase of apoptosis	*MTRNR2L2*<br>*MTRNR2L12*<br>*MTRNR2L8*	5.71E-06
		Cell adhesion	*L1CAM*<br>*GAS6*<br>*BCAM*<br>*ROBO3*	3.79E-02
		Regulation of tumor necrosis factor production	*CLU*<br>*GAS6*	1.67E-02
		Positive regulation of I-kappaB kinase/NF-kappaB signaling	*UBD*<br>*S100A4*	2.68E-02
