# Supplementary figures and images for: WWOX Loses the Ability to Regulate Oncogenic AP-2γ and Synergizes with Tumor Suppressor AP-2α in High-Grade Bladder Cancer
Source: Cancers (Basel). 2021 Jun 12;13(12):2957. doi: 10.3390/cancers13122957 (PMC8231628; doi:10.3390/cancers13122957)

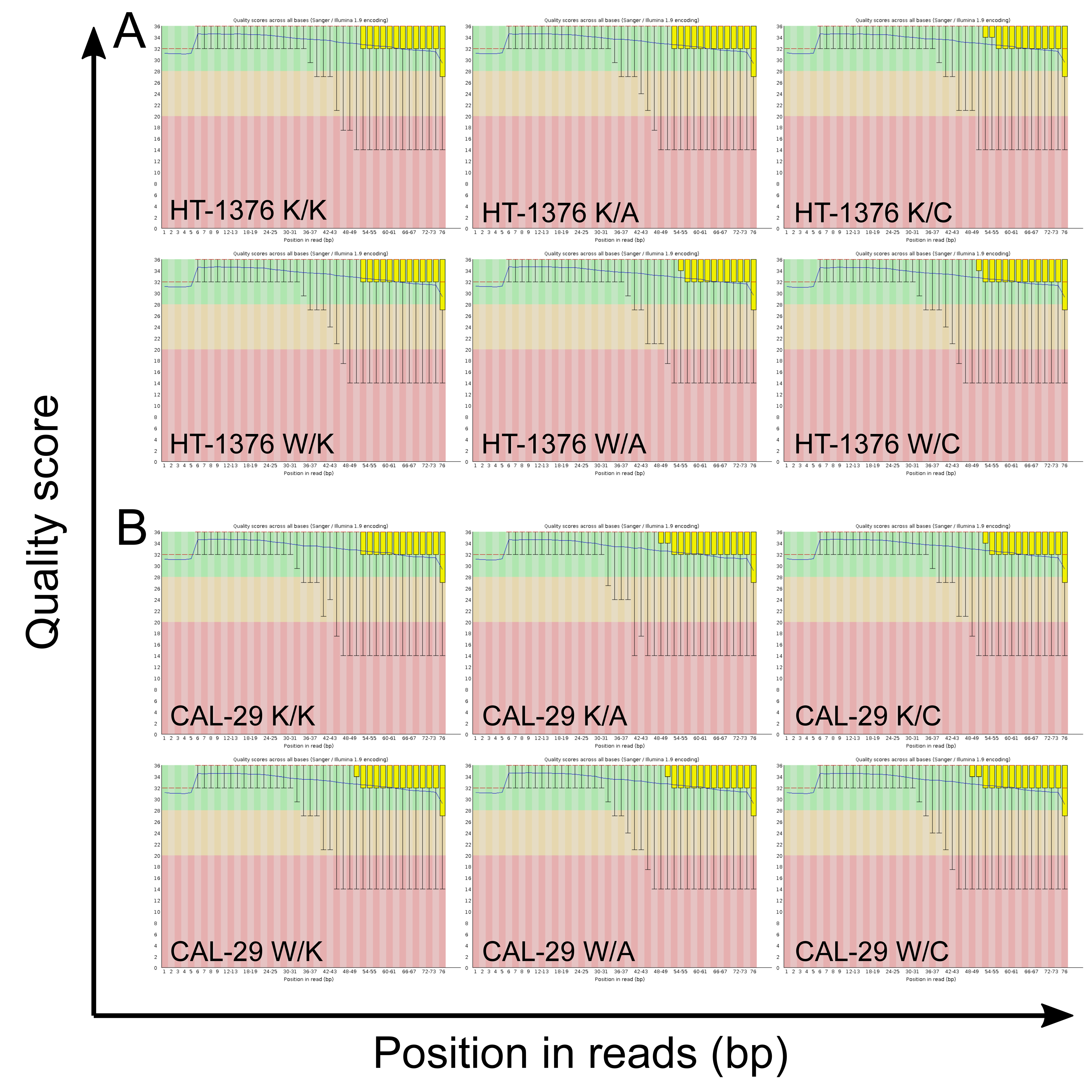

Supplement: Supplementary file 1 [file cancers-13-02957-s001.zip › Figure S1.tif]
